# Concomitant transcatheter edge-to-edge treatment for mitral regurgitation and the K-Clip system for tricuspid regurgitation: one case report

**DOI:** 10.3389/fcvm.2026.1791302

**Published:** 2026-06-08

**Authors:** Cai He, Hui Guo, Wenwen Chen, Wei Wang

**Affiliations:** Department of Cardiology, Wuhan Asia Heart Hospital, Wuhan, China

**Keywords:** K-Clip, mitral regurgitation, transcatheter edge-to-edge repair, transcatheter tricuspid valve interventions, tricuspid regurgitation

## Abstract

Transcatheter valve repair is an important alternative therapy for patients with severe valvular regurgitation who are at high risk for or contraindicated to undergo surgical procedures. However, there are challenges in bivalve interventional therapy. This case report presents a patient who underwent simultaneous transcatheter edge-to-edge repair (TEER) of the mitral valve and K-Clip™ tricuspid annuloplasty. The postoperative and one-month follow-up results were satisfactory, providing a new treatment approach for such patients.

## Introduction

Atrial dilated cardiomyopathy can lead to valve annular dilatation along with inadequate leaflet coaptation, inducing secondary mitral regurgitation (MR) and tricuspid regurgitation (TR). Currently, bivalvular interventional therapy faces significant challenges, particularly in concurrent dual-valve repair, which is technically more complex and associated with higher risks. We report a case of atrial dilated cardiomyopathy complicated with severe MR and TR. The patient underwent simultaneous mitral TEER and K-Clip™ tricuspid annuloplasty. This intervention significantly reduced the degree of valve regurgitation and improved the patient's quality of life.

## Case report

**Figure 1:**
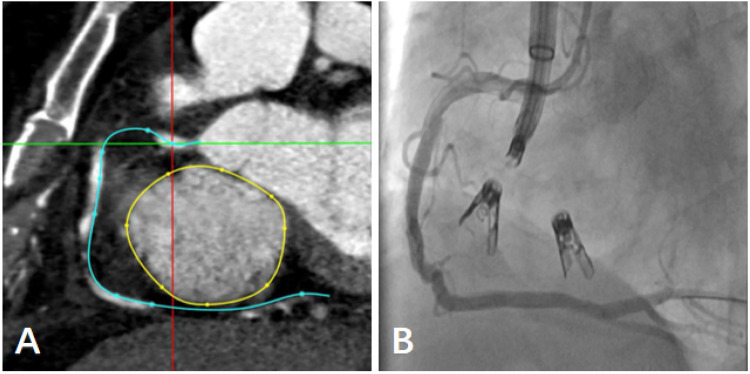
**(A)** The coronary CTA: the distance between the RCA and the tricuspid annulus is all greater than 3.0 mm. **(B)** After the tricuspid annuloplasty re-examination of right coronary angiography: TIMI grade 3 blood flow.

A 59-year-old female, height 161 cm, weight 78 kg, was admitted to Wuhan Asia Heart Hospital on November 8, 2025, due to intermittent chest tightness and shortness of breath. The patient had a history of hypertension and atrial fibrillation. Physical examination reveals jugular venous distension, and moist rales audible in the lower lung fields. The cardiac borders are enlarged, with a heart rate of 110 beats per minute and an irregular rhythm. Auscultation of the cardiac apex revealed a grade II/6 systolic murmur. Moderate edema is present in both lower extremities. Terminal pro-B-type natriuretic peptide was 3689.00 pg/mL. Transthoracic echocardiography (TTE): left atrium anteroposterior diameter of 58 mm, left ventricular end-diastolic dimension (LVEDd) of 66 mm, right atrial transverse diameter of 52 mm, right ventricular (RV) transverse diameter of 43 mm, severe MR (4+), severe TR (4+), left ventricular ejection fraction (LVEF) of 36%, pulmonary artery systolic pressure (PASP) of 60mmHg. Transoesophageal echocardiography (TEE): severe MR with a vena contracta width (VCW) of 0.9 cm. The proximal isovelocity surface area (PISA) method yields an effective regurgitant orifice area (EROA) of 0.58 cm^2^ and a regurgitant volume of 86 mL. The mitral valve orifice area is 7.4 cm^2^. The tricuspid valve has a three-leaf structure with uniform echoes. The annulus is dilated. The valve leaflets open fully during diastole but close poorly during systole. Severe TR (4+) with a VCW of 0.95 cm. Regurgitant orifices located at the anteroseptal, central, and posteroseptal positions. Annular dilatation is present, tricuspid anterior-posterior annular diameter of 42.5mm, tricuspid septolateral annular diameter of 43.1 mm. The PISA method yields an EROA of 0.76 cm^2^, a regurgitant volume of 120 mL, RV fractional area change (FAC) of 40%, Tricuspid annular systolic excursion (TAPSE) of 22 mm. The coronary CTA shows that the distance between the RCA and the tricuspid annulus is all greater than 3.0 mm, indicating that the risk of coronary artery compression during the operation is relatively low (Figure 1A).

Despite optimal medical therapy including diuresis and myocardial remodeling inhibition (Sacubitril/valsartan 25 mg bid, metoprolol 23.75 mg qd, spironolactone 20 mg qd, vericiguat 2.5 mg qd, dapagliflozin 10 mg qd), the patient exhibited persistent symptoms of heart failure. Consequently, the case presents clear indications for surgical intervention. The Society of Thoracic Surgeons (STS) score is 7.45%, indicating a high surgical risk. Combining the existing treatment methods, it was decided to perform mitral TEER and K-Clip™ tricuspid annuloplasty simultaneously.

**Figure 2:**
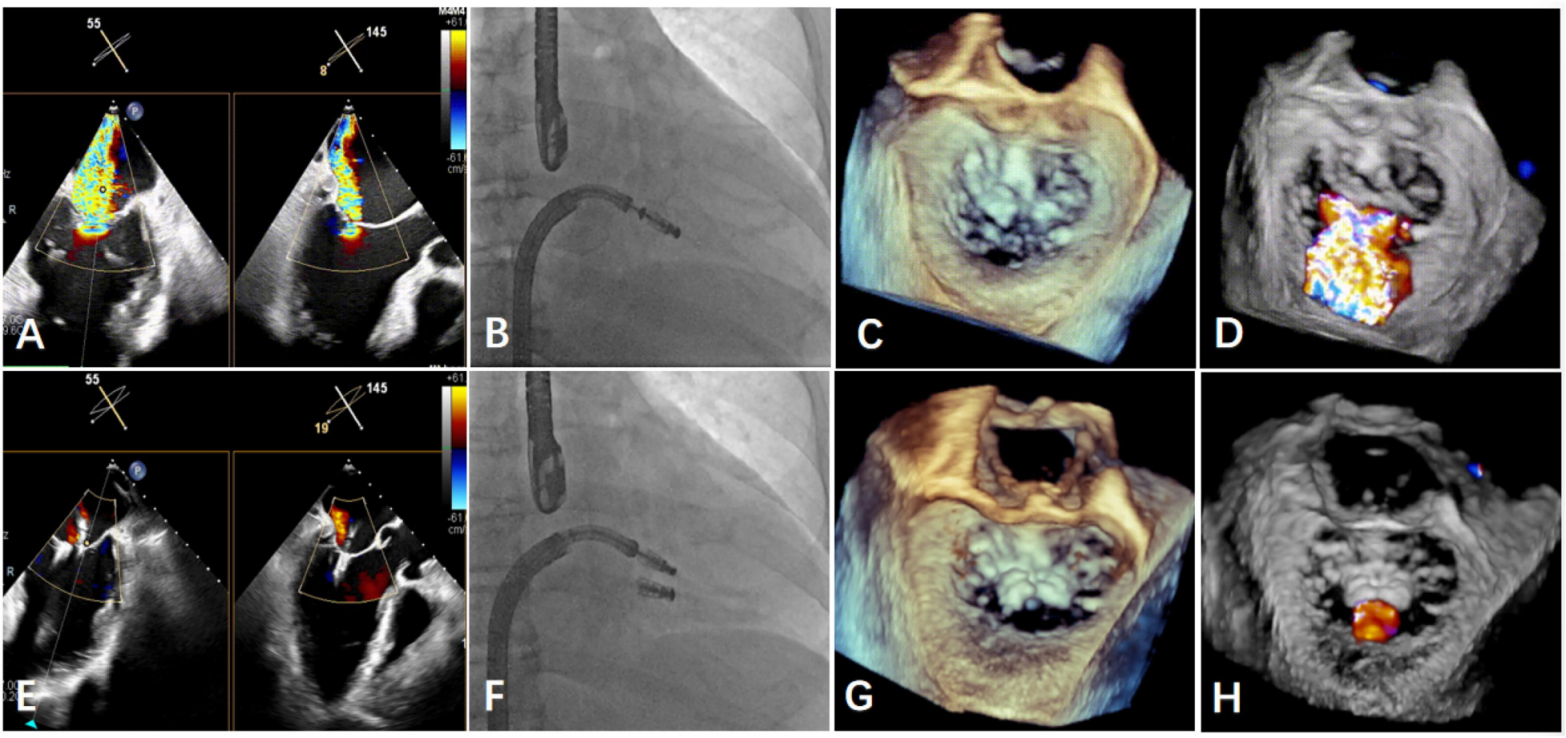
Pre- and post-operation images of the mitral TEER. **(A,E)** MR was reduced from severe(4+) to mild(1+) during systole in 2-dimensional TEE. **(B,F)** Position of the mitral valve clip under fluoroscopy. **(C,G)** Mitral valve morphology during systole in 3-dimensional TEE. **(D,H)** MR was reduced from severe(4+) to mild(1+) during systole 3-dimensional TEE-color flow.

**Figure 3:**
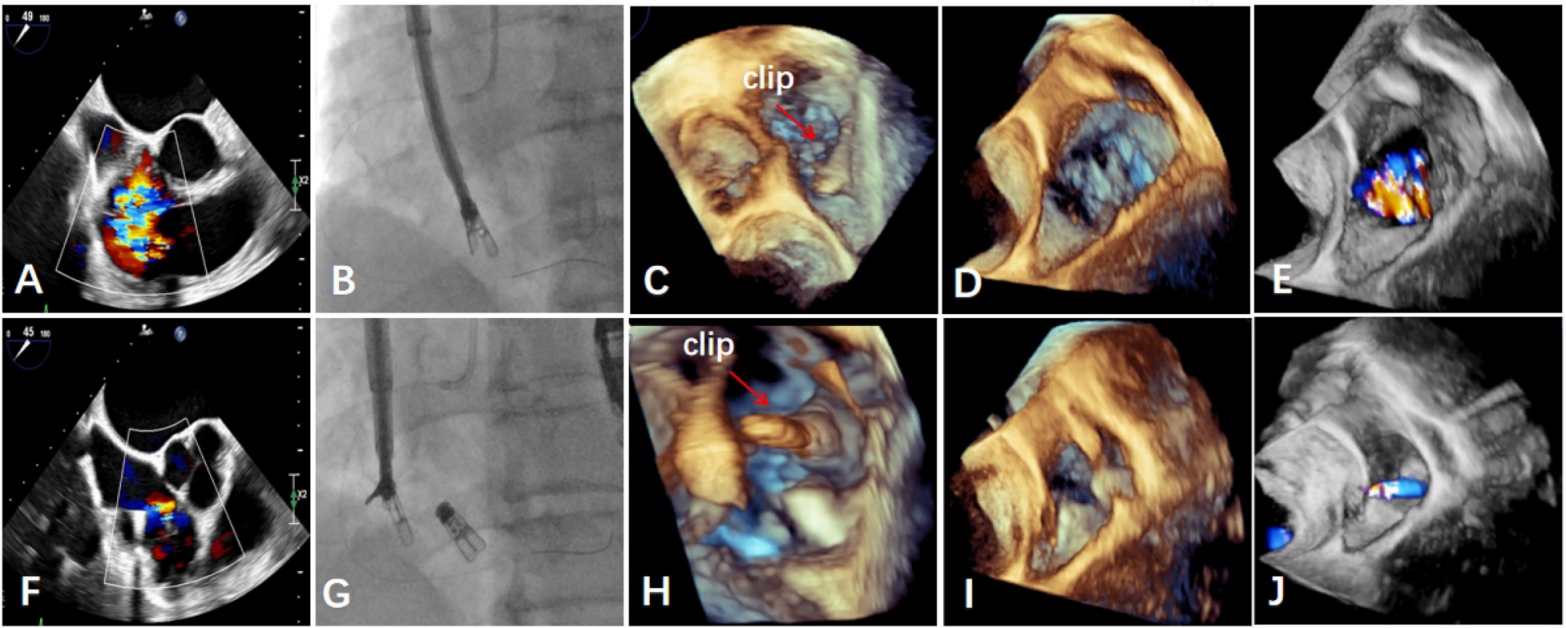
Pre- and post-annuloplasty images of the K-Clip tricuspid system. **(A,F)** TR was reduced from severe(4+) to mild(1+) during systole in 2-dimensional TEE. **(B,G)** Position of the tricuspid valve clip under fluoroscopy, **(C)** Anchor point 1: the junction of the posterior and septal leaflets, **(H)** Anchor point 2: the junction of the anterior and posterior leaflets; **(D,I)** Tricuspid valve morphology during systole in 3-dimensional TEE, **(E,J)** TR was reduced from severe(4+) to mild(1+) during systole in 3-dimensional TEE-color flow.

On November 13, 2025, a mitral TEER and K-Clip™ tricuspid annuloplasty were guided by echocardiography and fluoroscopy. The procedural details are outlined as follows:

1. M-TEER at 10:40: During the operation, a zipper-like strategy was adopted. A short-wide valve clip (Dejin Hangzhou Medical, China) was deployed in the central segment of the anterior leaflet of the mitral valve (A2) extending toward the medial segment of the anterior leaflet (A3) to reduce leaflet tension and mitigate the risk of laceration. After clamping, the tension of the posterior leaflet was acceptable, but there was still 2-3 + residual regurgitation on the left side of the clip. Subsequently, a second short-wide valve clip was implanted in the central segment of the anterior leaflet (A2) bordering the lateral segment of the anterior leaflet (A1). Following clip deployment, regurgitation was reduced to mild(1+) (Figure 2).
2. The K-Clip™ tricuspid annuloplasty began at 12:10 and ended at 14:00, lasting for 110 minutes. Based on the morphology of the tricuspid annulus and the location of the regurgitation orifice, a strategy involving the application of two K-Clip devices (Shanghai Huihe Medical, China) was implemented. Anchor point 1: 5:30 direction in the short-axis position of the tricuspid valve (clipping the junction of the posterior and septal leaflets), clip model: 12 T (36Fr). Anchor point 2: 7:30 direction in the short-axis position of the tricuspid valve (clipping the junction of the anterior and posterior leaflets), clip model: 14 T(42Fr). Intraoperative TEE indicated that tricuspid regurgitation was reduced from severe(4+) to mild(1+),the VCW measures 0.2 cm, tricuspid anterior-posterior annular diameter of 31mm, tricuspid septolateral annular diameter of 32 mm. The PISA method yields an EROA of 0.18 cm^2^, regurgitant volume of 28 mL, RV FAC of 41%, TAPSE of 21 mm (Figure 3). Re-examination of RCA angiography indicated TIMI grade 3 blood flow (Figure 1B)

**Figure 4:**
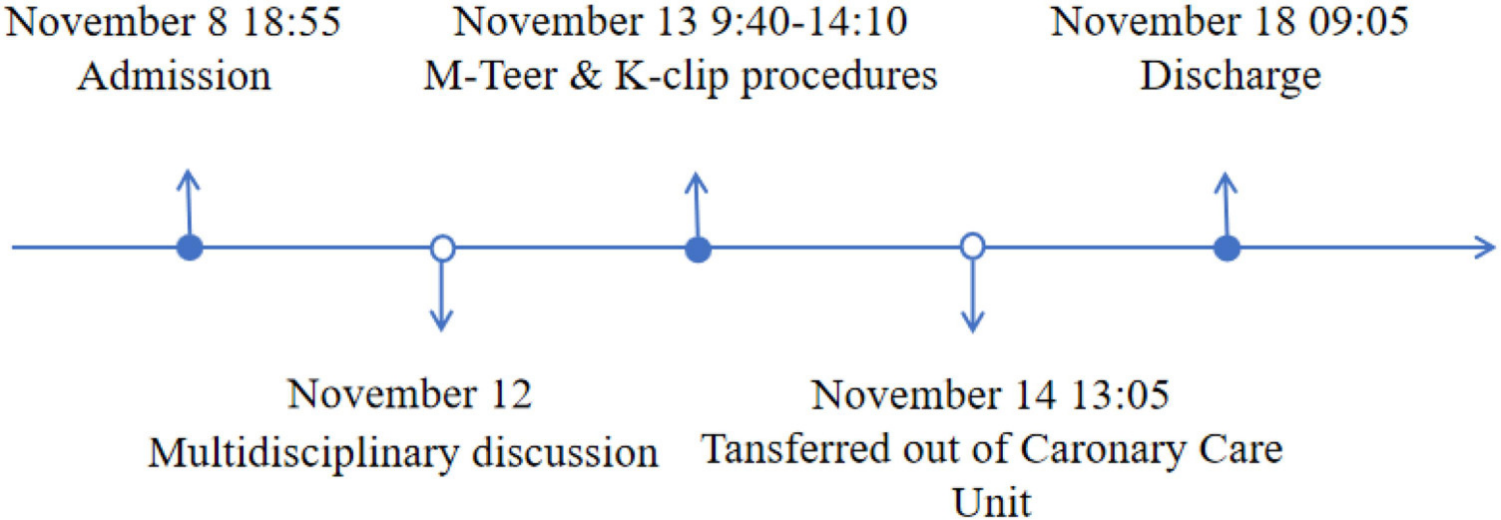
A timeline with relevant data from the episode of care.

The patient's vital signs were stable after the operation. Following day-1 ambulation was achieved, with hospital discharge on day 4 (Figure 4). At the one-month follow-up, the patient reported no discomfort. The TTE showed mild regurgitation of the tricuspid and mitral valves, left atrial anteroposterior diameter of 54 mm, LVEDd of 63 mm, RA left-right diameter 39 mm, RV left-right diameter 33 mm, LVEF of 45%.The adjustment of drug were as follows: Sacubitril/valsartan 25→50 mg bid, metoprolol 23.75→47.5 mg qd, the dosages of the other medications remained unchanged.

## Discussion

In this clinical case, we performed a “one-stop” interventional repair of the mitral and tricuspid valves for the patient, achieving a one-time solution to the problem of regurgitation in both valves. This effectively avoids the risk of aggravation of TR caused by fluctuations in LV function during the interval between staged surgeries, and reduces the risks associated with multiple anesthetics and repeated vascular punctures.

Approximately 90% of TR is functional, with half of it secondary to left heart disease. Among patients with functional mitral valve disease, 30% have moderate to severe TR (1, 2). TR is a recognized predictor of poor prognosis after surgical treatment of left heart valve disease. The tricuspid valve has long been underestimated. The previous notion was that TR could be alleviated or even disappear after successful treatment of the mitral valve. However, studies have shown that some patients still have persistent or progressive significant TR after mitral valve replacement or repair, which increases the risk of right heart failure and often makes symptoms difficult to control (3, 4). Given the risk of progressive aggravation of TR and RV failure, current guidelines recommend that for patients undergoing left heart valve surgery with severe TR, or those with mild to moderate TR but with annular dilation (≥ 40 mm) or signs of right heart failure, tricuspid valve (TV) surgery should be performed simultaneously (5). Compared with isolated mitral valve intervention, combined mitral and tricuspid valve treatment can improve the survival rate of patients and reduce the readmission rate (6–11). Therefore, for specific patients, simultaneous or staged combined surgery should be considered to optimize the treatment effect (12). Transcatheter tricuspid annuloplasty has emerged as an alternative therapy for the treatment of severe functional TR in patients at high risk for surgical intervention, demonstrating good safety and efficacy (13). Our patient has atrial dilated cardiomyopathy with significant dilation of both atria and annulus. It is considered that the main cause of TR is atrial functional. Even after mitral valve intervention, the possibility of improvement in tricuspid regurgitation is small. Therefore, we chose to intervene on both the mitral and tricuspid valves simultaneously.

**Figure 5:**
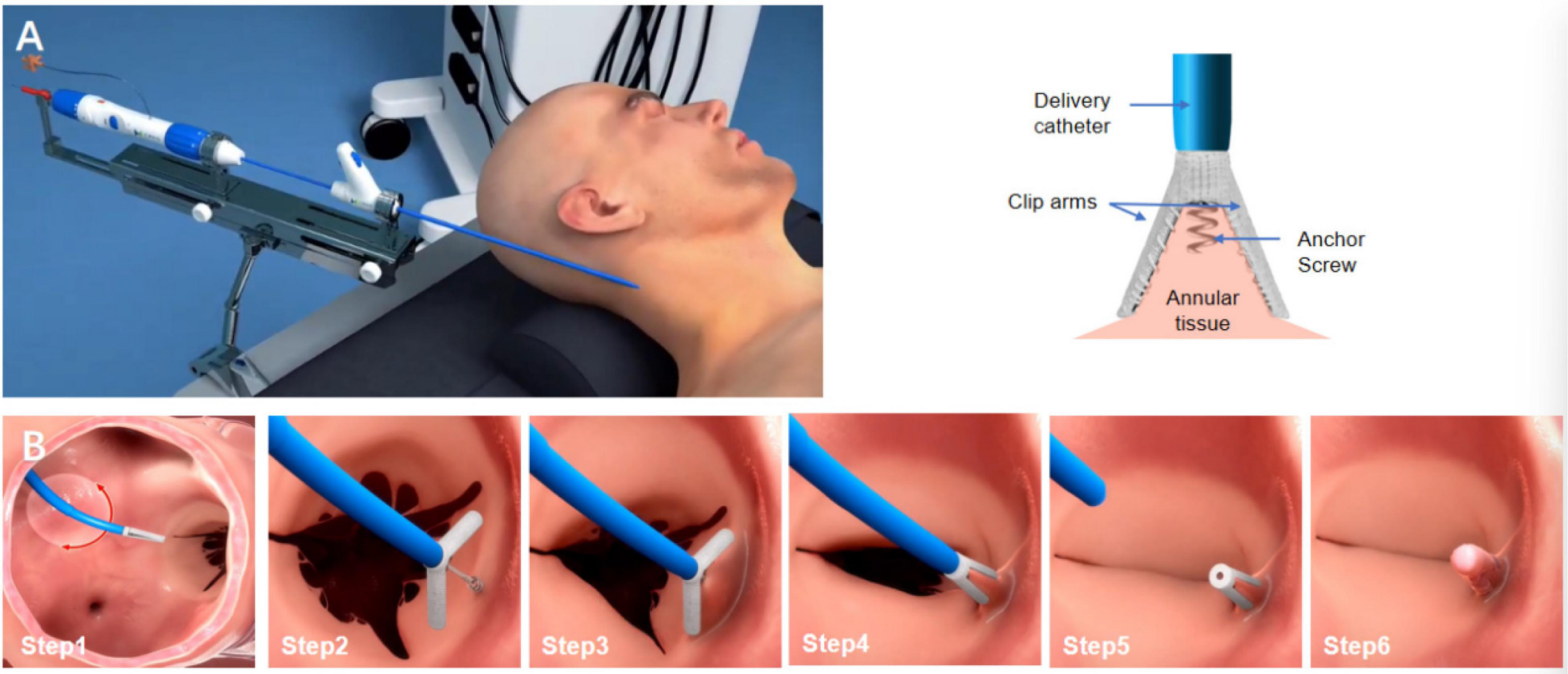
K-Clip® system. **(A)** K-Clip® system comprises two clip arms and a central anchoring screw, forming a “clip arm–annulus–screw sandwich” structure. Four clip sizes (arm length 36, 42, 48, 54Fr) are available. **(B)** K-Clip® operating Steps. Step 1: Through the jugular vein approach, enter the right atrium and adjust the delivery system to point towards the tricuspid annulus. Step 2: Drive the anchoring component into the target point on the annulus. Step 3: Adjust the grasping arms onto the annulus and gently lift the tissue. Step 4: Close the grasping arms to form a stable sandwich structure. Step 5: Detach the annuloplasty device and retract the delivery system. Step 6: After detachment, endothelialization occurs and the structure integrates as one.

K-Clip™ is a novel transcatheter tricuspid annuloplasty device first developed in China. It employs an innovative clip-based annular folding technique to reduce the size of the tricuspid annulus. The details of the K-Clip annuloplasty system used for implantation have been described in Figure 5. The postoperative anatomical structure of the tricuspid valve only undergoes slight deformation, which makes it possible to repair or replace it with other devices subsequently (13, 14). The preliminary clinical experience of the K-Clip tricuspid annuloplasty in patients with severe functional tricuspid regurgitation (FTR) has demonstrated good short-term efficacy and safety, including sustained reduction of TR, high survival rate, and low rehospitalization rate (15–17). Therefore, the device offers a new treatment option for patients with severe FTR.

Tricuspid annuloplasty achieves annular reduction by clamping the posterior and septal annulus of the tricuspid valve. In this area, the RCA runs along the atrioventricular groove with an extremely short anatomical distance. Excessive clamping depth or positional deviation can easily lead to compression, laceration or occlusion of the RCA. Herefore, it is of great significance to assess the distance between the RCA and the annulus through coronary CTA before the operation. A distance of less than 3 millimeters is considered a very high risk. During the operation and before the clip is fully released, coronary angiography should be performed on the RCA to rule out coronary artery compression. The area where the tricuspid annulus clamped is close to the atrioventricular node and the right bundle branch. The blood supply to the atrioventricular node mainly comes from the RCA. During the operation, mechanical compression, local edema or coronary artery ischemia may occur, which can lead to atrioventricular block and right bundle branch block. Current research data show that the incidence of conduction block is relatively low (16).

However, for combined valve diseases with different characteristics, more combinations of interventional methods are needed to ultimately achieve individualized treatment. Furthermore, the long-term efficacy of simultaneous dual-valve interventions necessitates large-scale cohort studies with extended follow-up durations.

## Conclusion

This case is the first reported instance of simultaneous TEER for MR and K-Clip™ for TR. Postoperatively, the regurgitation of both valves was significantly reduced, symptoms improved, and the quality of life was notably enhanced. It provides new ideas and approaches for patients with multiple valve diseases who are at high risk for surgical procedures.

## Data Availability

The raw data supporting the conclusions of this article will be made available by the authors, without undue reservation.
